# Role of *Bacillus licheniformis* VS16-Derived Biosurfactant in Mediating Immune Responses in Carp Rohu and its Application to the Food Industry

**DOI:** 10.3389/fmicb.2017.00514

**Published:** 2017-03-28

**Authors:** Sib Sankar Giri, Shib Sankar Sen, Jin Woo Jun, V. Sukumaran, Se Chang Park

**Affiliations:** ^1^Department of Biotechnology, Periyar Maniammai UniversityThanjavur, India; ^2^Laboratory of Aquatic Biomedicine, College of Veterinary Medicine and Research Institute for Veterinary Science, Seoul National UniversitySeoul, South Korea; ^3^School of Life Sciences, Jawaharlal Nehru UniversityNew Delhi, India

**Keywords:** *Bacillus licheniformis* VS16, biosurfactant, fish immune responses, immune-gene expression, disease resistance, heavy metal removal

## Abstract

Multifarious applications of *Bacillus licheniformis* VS16-derived biosurfactant were explored. *Labeo rohita* fingerlings were injected intraperitoneally with 0.1 mL of phosphate-buffered saline (PBS) containing purified biosurfactant at 0 (control), 55 (S55), 110 (S110), 220 (S220), or 330 (S330) μg mL^-1^ concentrations. Various immunological parameters and the expression of immune-related genes were measured at 7, 14, and 21 days post-administration (dpa). At 21 dpa, fish were challenged with *Aeromonas hydrophila* and mortality was recorded for 14 days. Immune parameters such as lysozyme levels (39.29 ± 2.14 U mL^-1^), alternative complement pathway (61.21 ± 2.38 U mL^-1^), and phagocytic activities (33.37 ± 1.2%) were maximum (*P* < 0.05) in the S220 group at 14 dpa; but immunoglobulin levels (11.07 ± 0.83 mg mL^-1^) were highest in the S220 group at 7 dpa, compared to that in controls. Activities of digestive enzymes (amylase, protease, and lipase) were higher (*P* < 0.05) in the S220 and S330 groups than in the control group. Regarding cytokine gene expression, pro-inflammatory cytokines (TNF-α and IL-1β) were down-regulated (*P* < 0.05) in the S220 and S330 groups. Expression of IL-10, TGF-β, and IKB-α were up-regulated in the S220 and S330 groups at 14 dpa, with the highest levels in the S220 group. The expression of NF-κB p65 and IKK-β were down-regulated in treatment groups, and were lowest (*P* < 0.05) in the S220 group. The highest post-challenge survival rate (72.7%) was recorded in S220 group. Further, the potential of this substance to inhibit biofilm formation, and heavy metal removal from vegetables were also evaluated. Biosurfactant was effective in inhibiting biofilm formation up to 54.71 ± 1.27%. Moreover, it efficiently removed cadmium (Cd) from tested vegetables such as carrot, radish, ginger, and potato, with the highest removal efficiency (60.98 ± 1.29%) recorded in ginger contaminated with Cd. Collectively, these results suggest that isolated biosurfactant could be used in the aquaculture industry, in addition to its potential application to the food industry.

## Introduction

‘Biosurfactants are amphipathic surface active molecules consists of hydrophilic and hydrophobic moieties that act by emulsifying hydrocarbons, increasing their solubilization, and subsequently rendering them available for microbial degradation’ (Donio et al., 2013). There are various classes of biosurfactants, among which the major classes are phospholipids, glycolipids, lipopolysaccharides, lipoproteins-lipopeptides, fatty acids, neutral lipids, flavolipids, the complete cell-surface itself, as well as those that are not fully characterized (Bodour et al., 2004). Microbial biosurfactants have numerous advantages over chemical surfactants, such as less toxic, higher biodegradability, environmentally compatible, higher foaming capability, high selectivity, specific activity at extreme pH, temperatures, and salinity. Also, it can be synthesized from renewable food sources (Bezza and Chirwa, 2015). Recently, biosurfactants have been utilized in many unconventional fields, including the enhancement of oil recovery to reduce environmental impact during extraction, remediation of water insoluble pollutants, the replacement of traditional synthetic surfactants in healthcare, cosmetics, soap, and paints, and in the production and application of drug delivery systems (Omudu et al., 2014). Biosurfactants also exhibit anti-microbial, anti-tumor, and anti-inflammatory properties (Perfumo et al., 2010). Bacterial genera such as *Bacillus, Pseudomonas, Halomonas, Acinetobacter, Arthrobacter, Rhodococcus*, and *Enterococcus* have been reported to produce various types of biosurfactants (Satpure et al., 2010). The genus *Bacillus* consists of versatile microbial species that exhibit considerable biosurfactant production and produce cyclic lipopeptides and lipoproteins that include surfactins, fengicins, lichenysins, and bacillomycin as the major types biosurfactants (Mukherjee and Das, 2005; Swaathy et al., 2014).

Properties of biosurfactants including emulsion formation and stabilization, as well as anti-adhesive and antimicrobial activities, make them suitable for applications in food processing and formulation. The addition of rhamnolipid surfactants was shown to improve dough stability, texture, volume, and conservation of bakery products (Nitschke and Costa, 2007). A bioemulsifier isolated from *Enterobacter cloacae* was demonstrated as a potential viscosity improvement agent of interest to the food industry (Iyer et al., 2006). *Streptococcus thermophilus* derived surfactant has been used for fouling control of heat-exchanger plates in pasteurizers (Busscher et al., 1996). Further, a *Pseudomonas fluorescens* surfactant showed potential as an inhibitor of corrosion in stainless steel (Dagbert et al., 2006). In addition to its application to the food industry, recent studies have demonstrated that administration of microbial biosurfactants enhances the immune responses and disease resistance of fish (Giri et al., 2016c; Rajeswari et al., 2016). Therefore, potential applications of microbial surfactants in aquaculture need to be further exploited.

“Aquaculture has emerged as one of the most promising and fastest-growing industries with total global production rising to 66.63 million tons in 2012 from 63.6 million tons in 2011” (FAO, 2014). However, intensive aquaculture practices result in stress, poor water quality, poor nutritional status, overcrowding, and sudden changes in temperature. Further, widespread industrial developments have caused deteriorated aquatic environments (Giri et al., 2016a). Typically, antibiotics and chemotherapeutics are used to control diseases in aquatic animals. However, the overuse of such compounds results in the environmental hazards, food safety problems, and development of drug-resistant pathogens (Austin and Austin, 2007). Although vaccination has been used as an alternative strategy, a single vaccine is generally effective against only one type of pathogen (Pasnik et al., 2005). Recently, the application of probiotics has also received attention in aquaculture. However, one concern regarding its uses is antagonism with other beneficial bacteria, the possible development of virulence traits through horizontal gene transfer, and pathogenicity to humans (Sharifuzzaman et al., 2011). The limitations of antibiotics, chemotherapeutics, vaccines, and probiotics suggest that aquaculture disease management should emphasize on harmless, preventative, and lasting methods.

Fish primarily depends on their innate immune system (Uribe et al., 2011). They have mechanisms to protect them against foreign substances such as pathogens and toxins, among others. Recently, bacterial secondary metabolites have received greater attention for disease control in fish aquaculture. Liu et al. (2011) demonstrated that secondary metabolite cyclo-(L-Pro-Gly), isolated from *Anoxybacillus flavithermus* SX-4 could enhance non-specific immunity and disease resistance in carp (*Cyprinus carpio*). Isolated secondary metabolites from the soil isolate *B. simplex* increased cytokine responses and disease resistance against *Aeromonas hydrophila* challenge in *C. carpio* (Wang et al., 2011). Recently, Rajeswari et al. (2016) demonstrated that phospholipid biosurfactant from *Staphylococcus hominis* significantly enhanced specific and non-specific immunity as well as disease resistance in *Oreochromis mossambicus*. Therefore, screening for bacterial biosurfactants (secondary metabolites) might lead to the identification of an eco-friendly method to strengthen the fish immune system.

Lipopeptide biosurfactant production by various bacterial strains in solid state culture has been reported in various studies (Das and Mukherjee, 2007; Kiran et al., 2010b, 2014; Zouari et al., 2014). We previously showed that *Bacillus subtilis* VSG4, isolated from tropical soil (Oviya et al., 2012), produces biosurfactant that stimulates immune responses and cytokine gene expression in rohu (*Labeo rohita*), in addition to enhancing resistance to *A. hydrophila* infection in carp (Giri et al., 2016c). In the present study, a biosurfactant-producing strain, *Bacillus licheniformis*, was isolated from food waste. The effects of the extracted biosurfactant on immune responses and cytokine gene expression in *L. rohita*, and on disease resistance in carp were investigated. Furthermore, applications of the biosurfactant to the food industry were also studied.

## Materials and Methods

### Isolation and Enumeration of Bacterial Colonies

Household food waste samples (mainly containing rice, vegetables, and fruits) were collected from Thanjavur city, Tamil Nadu, India, and serially diluted up to 10^-7^. One milliliter of sample from each dilution was spread-plated onto nutrient agar plates, which were incubated at 37°C for 48 h. Morphologically diverse colonies were selected and stored at 4°C for further studies.

### Screening of Potential Biosurfactant-Producing Bacteria

The following methods were adopted to screen potential biosurfactant-producing strains: (i) drop-collapse test, by adding mineral oil to 96-well microtiter plates (Rodrigues et al., 2006); (ii) oil displacement activity, by adding weathered crude oil (Anandaraj and Thivakaran, 2010); (iii) emulsification activity, by adding kerosene and an equal volume of cell-free supernatant (Deepak and Jayapradha, 2015). The most efficient biosurfactant-producing strain was selected for further studies.

### Identification of Bacterial Strain

The morphological and biochemical characterization of the selected biosurfactant-producing strain was performed according to Bergey’s Manual of Determinative Bacteriology, 9th edition (Holt et al., 1994). Molecular identification was performed by 16S rRNA gene sequencing. Wizard^®^ genomic DNA purification kit (Promega, Madison, WI, USA) was used for genomic DNA extraction. PCR amplification and sequencing of the 16S rRNA gene were carried out using universal primers (5′-GAGTTTGATCCTGGCTCAG-3′; 5′-AGAAAGGAGGTGATCCAGCC-3′). Sequences were compared to those of other 16S RNAs obtained from GenBank using the BLAST program.

### Biosurfactant Production and Characterization

The screened isolate was inoculated in 25 mL of sterile nutrient broth and incubated at 37°C for 24 h at 300 rpm. Biosurfactant production was carried out according to the method described earlier (Giri et al., 2016c). Briefly, 10 mL of inoculum was transferred to a 1-L conical flask containing 500 mL of previously described medium (Joshi et al., 2013) and incubated at 37°C, pH 7.0, and 300 rpm for 72 h. After adjusting the pH to 7.0, cultures were centrifuged at 11300 × *g* at 4°C for 20 min to obtain cell-free supernatants. The supernatant was precipitated by adding 6 N HCl and pH was adjusted to 2.0. The collected precipitate (11300 × *g* at 4°C for 20 min) was dissolved in 5 mL of distilled water and pH was adjusted to 7.0 with 1 N NaOH. The biosurfactant solution was dialysed against demineralised water at 4°C using a Cellu-Sep© membrane (Seguin, USA) for 48 h. The partially purified biosurfactant was freeze-dried and stored at -20°C until use.

Initial identification of biosurfactant was performed using the phosphate (Okpokwasili and Ibiene, 2006) and biuret (Feigner et al., 1995) tests. Next, the partially purified biosurfactant was analyzed using a gas chromatography–mass spectrometry (GC–MS) system (Shimadzu QP2010 Plus, Japan) equipped with a capillary column (DB-5 MS; 0.25-mm film thickness, 0.25 mm i.d., 30 m length) (Giri et al., 2016c). “Briefly, helium was used as the carrier gas with a flow rate of 1.0 mL min^-1^. The injection port temperature was 250°C. The column oven temperature was held at 80°C for 2 min and then the temperature was increased at every 10°C min^-1^ up to 250°C, after which the temperature was finally raised at a rate of 5°C min^-1^ to 280°C; this temperature was held for 10 min. Electron impact spectra were acquired in positive ionization mode between m/z 40 and 450″ (Giri et al., 2016c).

The (cetyltrimethylammonium bromide) CTAB agar test (Siegmund and Wagner, 1991) was used to identify the type of surfactant, and the orcinol test (Chandrasekharan and Bemiller, 1980) was performed for the detection of reducing sugars. To estimate protein concentration, a Lowry test (Lowry et al., 1951) was performed. In addition, a phosphate test was performed to check for the presence of a phosphate moiety (Okpokwasili and Ibiene, 2006).

### Immunomodulatory Activities of Biosurfactant in *L. rohita*

#### Experimental Plan

*Labeo rohita* fingerlings (body weight: 21.47 ± 1.06 g) procured from a local fish farm were acclimatized to laboratory conditions for 2 weeks in 500-L plastic tanks at 26 ± 2°C. Fish were fed the basal diet (Giri et al., 2016c) twice a day at a rate of 2% of their body weight. Basic physicochemical parameters of the water were measured weekly (American Public Health Association [APHA] et al., 2012). This study was conducted following the “Guidelines on the Regulation of Scientific Experiments on Animals,” CPCSEA, GoI, and the experimental protocols were approved by the Institutional Ethics Committee of the Periyar Maniammai University.

The LC_50_ value of the biosurfactant in *L. rohita* was determined by acute toxicity testing as per the standard method (American Public Health Association [APHA] et al., 2012; Misik et al., 2012), starting with range-finding tests for acute toxicity trials. The 96-h LC_50_ of the biosurfactant in *L. rohita* was calculated as 2,650 μg mL^-1^ using the Probit method (Finney, 1980). Sub-lethal concentrations (55–330 μg mL^-1^) were used in this study.

The fish were randomly distributed into five experimental groups, with three replicates in each group. Each tank contained 35 fish (i.e., per group: 35 × 3 = 105 fish) and tank capacity was 200 L. First four fish groups were injected intraperitoneally (i.p.) with 100 μL of PBS containing the surfactant at concentrations of 55, 110, 220, and 330 μg mL^-1^, and these groups were labeled ‘S55,’ ‘S110,’ ‘S220,’ and ‘S330,’ respectively. The fifth group was injected with 100 μL of PBS and considered as control. The fish were fed the basal diet and kept for observation until 21 days.

#### Sample Collection and Immunological Parameters

Nine fish (3 × 3 = 9 fish per group) were randomly collected from each group at 7, 14, and 21 days post administration (dpa). ‘Blood samples were collected from the caudal vein using a 2-mL syringe after anesthetisation with MS222 (Sigma-Aldrich, St. Louis, MO, USA) and transferred to centrifuge tubes (Eppendorf, Germany).’ Serum was collected by centrifugation (4,000 × *g*, 10 min, 4°C) and stored at 4°C until use’ (Giri et al., 2015). Intestinal samples from the fish were dissected aseptically, rinsed with cold distilled water, and used for the analysis of intestinal enzyme activities.

Head kidney macrophages were isolated (3 × 3 = 9 fish per group) according to the method described by Li et al. (2013). Trypan blue exclusion test was used to evaluate the cell viability and cell concentration was measured using a haemocytometer. Harvested cells were adjusted to a concentration of 1 × 10^7^ cells mL^-1^ for further use.

##### Immune parameters

Serum lysozyme activity (LA) and alternative complement pathway (ACP) activity was determined according to the methods described by Ellis (1990) and Yano (1992), respectively Phagocytic activity (PA) of head kidney macrophages was determined using the method descried by Geng et al. (2012). The number of phagocytic cells per 100 adherent cells was microscopically determined. The PA was calculated following the formula: PA = (phagocytic leucocytes/total leucocytes) × 100. Serum bactericidal activity against *A. hydrophila* was tested as described by Welker et al. (2007). Serum immunoglobulin (IgM) levels were measured according to a previously described method (Giri et al., 2016b).

##### Intestinal Enzyme Activities

Amylase activity was assayed by the method described by Furné et al. (2005); protease activity was assayed as described by Anson (1938); lipase activity was determined using the method reported by Mckellar and Cholette (1986). All enzymatic activities were expressed as specific activity (U mg^-1^ protein).

#### Gene Expression Analysis

The head kidney from nine fish per group was dissected at 7, 14, and 21 dpa. “Total RNA was extracted using TRIzol reagent (Invitrogen, USA) according to the manufacturer’s instructions. Total RNA concentration and purity were determined using a spectrophotometer, and the quality was checked by agarose gel electrophoresis. The extracted RNA was treated with RNA-Free DNase (Takara, Shiga, Japan) to remove contaminating DNA and then reverse transcribed into cDNA using a Superscript cDNA synthesis kit (Life Technologies) following the manufacturer’s instructions” (Giri et al., 2015). Real-time PCR analysis of TNF-α, IL-1β, IL-10, IL-12, TGF-β, NF-κB p65, IKK-β, IκB-α, and β-actin mRNA expression was performed using the CFX96^TM^ Real-Time PCR platform (Bio-Rad) following standard protocols with the primers and thermo-cycling conditions indicated in **Table 1**. Carp specific primers were used in this study, and the sequences were selected from the literature (Giri et al., 2016c). “All PCRs were performed at least three times. To verify the accuracy of each reaction, we performed melting curve analysis and checked the dissociation curve after amplification. β-Actin was used as an endogenous control for data analysis. All samples were run in parallel with the housekeeping gene, which was used to normalize cDNA loading” (Giri et al., 2015). Gene expression results were analyzed using the 2^-ΔΔCT^ method after verification that the primers amplified with an efficiency of approximately 100% (Livak and Schmittgen, 2001); data for all treatment groups were compared to those of the control group.

**Table 1:** Real-time primer sequences and thermocycling conditions.

Target gene	Primer sequence (5′–3′)	Thermocycling conditions	Reference
TNF-α	CTCAACAAGTCTCAGAACAATCAGG TCCTGGTTCCTTCTCCAATCTAGCT	95° C 30 s, 40 cycles of 95°C 5 s, 61.1°C 30 s, and 72°C 30 s	Giri et al., 2016c
IL-1β	ATCTTGGAGAATGTGATCGAAGAG GATACGTTTTTGATCCTCAAGTGTGAAG	95° C 30 s, 40 cycles of 95°C 5 s, 61.1°C 30 s, and 72°C 30 s	Giri et al., 2016c
IL-10	AAGGAGGCCAGTGGCTCTGT CCTGAAGAAGAGGCTCTGT	95°C 30 s, 40 cycles of 95°C 5 s, 61.1°C 30 s, and 72°C 30 s	Giri et al., 2016c
TGF-β	ACGCTTTATTCCCAACCAAA GAAATCCTTGCTCTGCCTCA	95° C 30 s, 40 cycles of 95°C 5 s, 60.5°C 30 s, and 72°C 30 s	Giri et al., 2016c
NF-κBp65	TATTCAGTGCGTGAAGAAG TATTAAAGGGGTTGTTCTGT	95°C 30 s, 40 cycles of 95°C 5 s, 58°C 30 s, and 72°C 30 s	Giri et al., 2016c
IκB-α	TCTTGCCATTATTCACGAGG TGTTACCACAGTCATCCACCA	95° C 30 s, 40 cycles of 95°C 5 s, 62.3°C 30 s, and 72°C 30 s	Giri et al., 2016c
IKKβ	GTGGCGGTGGATTATTGG GCACGGGTTGCCAGTTTG	95°C 30 s, 40 cycles of 95°C 5 s, 60.3°C 30 s, and 72°C 30 s	Giri et al., 2016c
β-actin	AGACCACCTTCAACTCCATCATG TCCGATCCAGACAGAGTATTTACGC	95° C 30 s, 40 cycles of 95°C 5 s, 60.5°C 30 s, and 72°C 30 s	Giri et al., 2016c

#### Pathogen Challenge Test

At the end of the 21-days trial, 36 fish from every group (including the control group) were injected with 100 μL of PBS containing 1 × 10^7^ live *A. hydrophila*. The dose was determined based on a preliminary study. For negative control, another 30 fish (fed on a basal diet during the trial) were injected with 100 μL of PBS. The daily mortality of the challenged fish was recorded for 14 days. The relative percentage survival (RPS, %) was calculated using the formula of Amend (1981) as follows: RPS = (1- % mortality in treated group/% mortality in control group) × 100.

### Heavy Metal Removal from Vegetables

Vegetables such as potato, carrot, radish, and ginger were collected from the local market of Thanjavur, Tamil Nadu. Vegetables were washed thoroughly with running tap water for 30–40 min and then washed with double distilled water. The skins were removed, cut into small pieces, immersed in a 1:1 HCl solution for 10 min, and then washed with double deionised water (Anjum et al., 2016).

Cadmium chloride stock solution was prepared in milli-Q water at a concentration of 1000 mg L^-1^. A violet color developed when diphenylcarbazide was added and the absorbance was then determined at 540 nm using a UV-VIS spectrometer and a standard curve was plotted.

Vegetables were exposed to cadmium chloride at concentrations of 0.3, 0.4, and 0.5 mg mL^-1^ for 30 min and diphenylcarbazide was added to develop the violet color (Anjum et al., 2016). The absorption of the Cd ion concentration was determined using a UV-VIS spectrophotometer. Few of the vegetables from the same stock were treated with biosurfactant; after the absorption with cadmium chloride, the change in concentration was again determined by measuring the OD at 540 nm. The cadmium ion removal percentage due to bio-adsorption was calculated as: percent Cd removal = (C_i_ – Co/C_i_) × 100, where C_i_ = initial concentration of cadmium (mg L^-1^) and Co = final concentration of cadmium (mg L^-1^).

### Biofilm Inhibition Assay

Biofilm inhibition assay was performed following the previously described method of Anjum et al. (2016) with slight modification. Overnight culture of *Escherichia coli* was prepared in 96-well plates containing 100 μL LB medium per well (37°C, 200 rpm). After 20 h, a 96-pin replicator (Boekel Scientific) was applied to inoculate 96-well test plates containing 150 μL of relevant pre-heated medium. To avoid the evaporation of medium, test plates were transferred to large plastic bags and incubated at 37°C for 48 h without shaking. After 48 h, biofilm was formed in a 96-well microtiter plate. The supernatant was discarded and the wells were washed with PBS to remove non-adherent cells. Then, the biofilm was treated with biosurfactant along with the control. The plates were incubated at 37°C for 2 h and 4 h. Subsequently, the plates were washed twice to remove non-adherent cells. A 100-μL solution of 0.4% crystal violet was added, incubated for 30 min, and washed with PBS to remove the extra stain. The biofilm was air dried for 5 min, then absolute ethanol was added to solubilize the crystal violet. The optical density was determined using a spectrophotometer (Perkin-Elmer, USA) at 590 nm.

### Statistical Analysis

Analysis of variance (ANOVA) tests were used to analyze the data. A Tukey’s test was used to analyze differences between the treatments. The OriginPro software (version 8; OriginLab Corporation, Northampton, MA, USA) was used for statistical analysis. The significance level fixed at *P <* 0.05. Results were expressed as the mean ± SEM.

## Results

### Isolation and Screening of Biosurfactant-Producing Bacteria

Eighteen different bacterial colonies were selected from nutrient agar plates, of which eight isolates exhibited positive haemolysis activity. Among them, strain VS16 exhibited the highest haemolytic activity in terms of zone of clearance (2.97 cm radius) on blood agar. Emulsion formation is the stable interaction between the hydrophobic and hydrophilic phases. Among the isolates, VS16 had highest emulsification index of 49.03% during the tropophase and hence was selected for further studies. Results of the drop collapse and oil displacement tests confirmed that VS16 is highly capable of producing biosurfactant. Hence, VS16 was used for further studies.

Strain VS16 consisted of gram-positive, rod shaped bacteria that were arranged as chains. The results of biochemical tests indicated that the isolate was similar to *Bacillus* spp. Phenotypic identification was confirmed by 16S rRNA gene sequence analysis. BLAST search analysis^1^ of the obtained 16S rRNA sequence (629 bp) showed high identity scores when compared to sequences from the *Bacillus* genus. Strain VS16 had the highest nucleotide sequence similarity value (99%) when compared to that of *B. licheniformis* (GenBank accession numbers: KJ526872.1, KF737353.1, AY842876.1, and AY859479.1) (Phylogenetic data not shown). The nucleotide sequence of this strain has been submitted to NCBI GenBank under the accession number KX943303.

### Biosurfactant Production and Characterization

The result of CTAB showed no coloration of colonies or of CTAB agar, which indicates that the biosurfactant was not a glycolipid. Based on the Orcinol test, the expected red-orange coloration was not observed, indicating the absence of reducing sugars in the biosurfactant. The partially purified biosurfactant tested positive using Lowry’s test, thus indicating the presence of a lipopeptide moiety. Moreover, a phosphate assay confirmed its phospholipid type. These results demonstrated that the *B. licheniformis* VS16-derived biosurfactant could be a type of phospho-lipopeptide biosurfactant.

**FIGURE 1:**
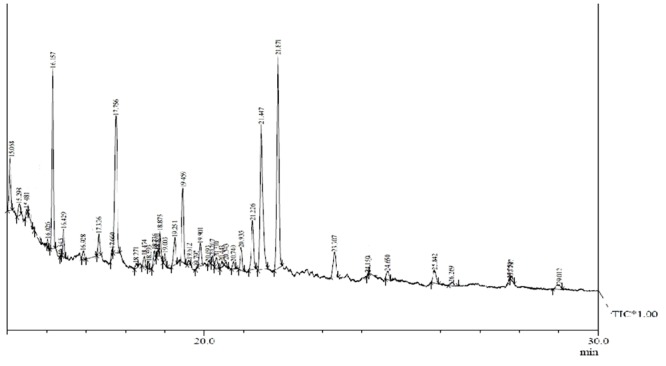
**Gas chromatography–mass spectrometry (GC–MS) analysis of partially purified *Bacillus licheniformis* VS16-derived biosurfactant**.

Gas chromatography–mass spectrometry analysis (**Figure 1** and **Table 2**) revealed the presence of 11 lipid-based compounds with biosurfactant properties. Major peaks at retention times of 16.15, 17.75, 21.44, and 21.87 min were confirmed as eicosanoic acid, heptacosane, tert-hexadecanoic acid, and hexadecanoic acid, with molecular weights of 326, 380, 258, and 270 Da, respectively. Similarly, heneicosane, octadecanoic acid and methyl ester, myristoleic acid, and pentadecanoic acid were identified at the retention times of 19.25, 19.45, 20.93, and 21.22 min with molecular weight (Da) of 296, 298, 226, and 242, respectively. Peaks at 15.06, 23.30, and 25.84 min were confirmed as 9-hexadecenoic acid, methyl ester, myristic acid, methyl ester, and mannosamine with molecular weights of 254, 232, and 215.6, respectively. The highest percentage of peak area was for hexadecanoic acid and the lowest percentage peak (2.91%) was identified for myristoleic acid at a retention time of 20.93 min.

**Table 2:** Compounds identified from *Bacillus licheniformis* VS16 derived biosurfactant by gas chromatography–mass spectrometry (GC–MS) analysis.

Sl no	Retention time	Name of the compounds	Molecular formula	Molecular weight (Da)	Peak area %
1	15.06	9- Hexadecenoic acid, methyl ester	C_16_H_30_O_2_	254	4.17
2	16.15	Eicosanoic acid, methyl ester	C_21_H_42_O_2_	326	13.62
3	17.75	Heptacosane	C_27_H_56_	380	14.3
4	19.25	Heneicosane	C_21_H_44_	296	2.40
5	19.45	Octadecanoic acid, methyl ester	C_19_H_38_O_2_	298	5.87
6	20.93	Myristoleic acid	C_14_H_26_O_2_	226	2.132
7	21.22	Pentadecanoic acid	C_15_H_30_O_2_	242	4.74
8	21.44	Tert-hexadecanoic acid	C_16_H_34_S	258	18.16
9	21.87	Hexadecanoic acid, methyl ether	C_17_H_34_O_2_	270	21.76
10	23.30	Myristic acid, methyl ester	C_14_H_28_O_2_	232	2.91
11	25.84	Mannosamine	C_6_H_13_NO_5_:HCl	215.6	1.79

### Effect of Biosurfactant on Immune Responses in *L. rohita*

The highest LA and ACP activities (*P* < 0.05) were recorded 14 days in S220 group (**Table 3**). However, both LA and ACP activities slightly declined at 21 days, when compared to those recorded at 14 days, except in the S55 group.

**Table 3:** Lysozyme activity (LA) and alternative complement pathway (ACP) activities in *Labeo rohita* administered (i.p) with purified biosurfactant.

Group	LA (U mL^-1^)			ACP (ACH_50_U mL^-1^)		
	7 days	14 days	21 days	7 days	14 days	21 days
Control	28.31 ± 1.04^a^	28.79 ± 1.13^a^	28.46 ± 0.81^a^	47.31 ± 1.96^a^	48.06 ± 2.11^a^	47.83 ± 1.73^a^
55 μg mL**^-^**^1^	28.38 ± 0.93^a^	29.94 ± 1.02^ab^	31.87 ± 1.14^ab^	49.14 ± 2.10^ab^	48.68 ± 1.86^ab^	49.73 ± 1.38^a^
110 μg mL**^-^**^1^	32.96 ± 1.64^b^	34.61 ± 1.72^bc^	31.97 ± 0.86^ab^	50.57 ± 2.48^ab^	54.37 ± 1.73^bc^	53.11 ± 2.06^bc^
220 μg mL**^-^**^1^	35.83 ± 1.27^bc^	39.29 ± 2.14^c^	35.02 ± 1.82^b^	53.78 ± 1.82^b^	61.21 ± 2.38^c^	56.47 ± 1.62^c^
330 μg mL**^-^**^1^	37.92 ± 1.39^c^	34.98 ± 1.62^bc^	32.65 ± 1.37^ba^	54.19 ± 1.93^b^	57.02 ± 1.57^c^	54.71 ± 2.14^c^

*Values in the same column with different superscripts letters are significantly different (*P* < 0.05). Values are presented as mean ± SEM (*n* = 9).*

Significantly higher PA was recorded in the S220 and S330 groups than in the control group at all time points (**Table 4**), with highest in the S220 group at 14 days. IgM levels were higher in the treated groups (**Table 4**). Fish in the S220 group exhibited significantly higher IgM levels at all time points, with a highest in the S220 group at 7 days (**Table 4**).

**Table 4:** Phagocytic activity (PA) and immunoglobulin M (IgM) activities in *L. rohita* administered (i.p) with purified biosurfactant.

Group	PA (%)			IgM (mg mL^-1^)		
	7 days	14 days	21 days	7 days	14 days	21 days
Control	17.26 ± 0.47^a^	17.84 ± 0.52^a^	16.93 ± 0.39^a^	6.82 ± 0.41^a^	6.78 ± 0.53^a^	6.91 ± 0.38^a^
55 μg mL**^-^**^1^	18.64 ± 0.72^ab^	19.23 ± 0.31^a^	18.82 ± 0.26^a^	7.63 ± 0.48^a^	8.11 ± 0.36^ac^	8.37 ± 0.62^ab^
110 μg mL**^-^**^1^	22.73 ± 0.81^bc^	26.18 ± 0.59^b^	22.96 ± 0.74^ac^	8.16 ± 0.37^ab^	9.68 ± 0.71^bc^	9.48 ± 0.43^b^
220 μg mL**^-^**^1^	25.18 ± 0.62^c^	33.37 ± 1.20^c^	28.13 ± 1.3^bc^	11.07 ± 0.83^b^	10.57 ± 0.62^b^	8.96 ± 0.72^bc^
330 μg mL**^-^**^1^	26.74 ± 1.03^c^	30.24 ± 0.79^bc^	26.98 ± 1.07^c^	10.74 ± 0.52^b^	8.62 ± 0.92^ba^	6.93 ± 0.46^ac^

*Values in the same column with different superscripts letters are significantly different (*P* < 0.05). Values are presented as mean ± SEM (*n* = 9).*

### Effect of Biosurfactant on Digestive Enzyme Activities in *L. rohita*

Administration (i.p.) of biosurfactant enhanced digestive enzyme activities in fish (**Table 5**). Amylase activity was significantly higher in the S220–S330 groups at 14 and 21 dpa than in the controls, and was highest in the S220 group at 14 dpa. Protease activity was significantly higher in the S220 group at 14 and 21 dpa with the highest levels at 14 dpa. Lipase activity was higher (*P* < 0.05) in the S220 and S330 groups at 14 and 21 dpa (**Table 5**); however, highest lipase activity was recorded in the S330 group at 7 dpa.

**Table 5:** Digestive enzyme activities of *L. rohita* administered with biosurfactant.

Parameters	Doses of biosurfactant	Enzyme activities		
		7 days	14 days	21 days
Amylase	0 μg mL**^-^**^1^	7.03 ± 0.13^a^	7.06 ± 0.08^a^	7.08 ± 0.11^a^
	55 μg mL^-1^	7.07 ± 0.10^a^	7.14 ± 0.09^ab^	7.21 ± 0.13^a^
	110 μg mL^-1^	8.26 ± 0.17^ab^	9.82 ± 0.14^b^	7.89 ± 0.11^ab^
	220 μg mL^-1^	8.86 ± 0.21^ab^	11.68 ± 0.19^b^	9.17 ± 0.24^b^
	330 μg mL^-1^	10.31 ± 0.26^b^	10.54 ± 0.32^b^	9.23 ± 0.18^b^
Protease	0 μg mL^-1^	2.03 ± 0.04^a^	2.07 ± 0.08^a^	2.06 ± 0.05^a^
	55 μg mL^-1^	2.08 ± 0.04^a^	2.10 ± 0.09^a^	2.27 ± 0.06^ab^
	110 μg mL^-1^	2.41 ± 0.06^a^	3.04 ± 0.14^ab^	2.79 ± 0.08^ab^
	220 μg mL^-1^	2.69 ± 0.05^a^	4.36 ± 0.19^b^	3.73 ± 0.04^b^
	330 μg mL^-1^	2.27 ± 0.07^a^	3.14 ± 0.12^b^	2.43 ± 0.10^ab^
Lipase	0 μg mL^-1^	4.83 ± 0.08^a^	4.91 ± 0.06^a^	5.04 ± 0.06^a^
	55 μg mL^-1^	4.97 ± 0.06^a^	5.12 ± 0.05a	5.26 ± 0.08^a^
	1l0 μg mL^-1^	5.29 ± 0.09^ab^	5.39 ± 0.06^ab^	5.44 ± 0.05^a^
	220 μg mL^-1^	5.41 ± 0.11^ab^	6.32 ± 0.08^b^	5.36 ± 0.07^a^
	330 μg mL^-1^	6.57 ± 0.08^b^	6.28 ± 0.09^b^	5.23 ± 0.08^a^

### Effect of Biosurfactant on Cytokine Gene Expression

**FIGURE 2:**
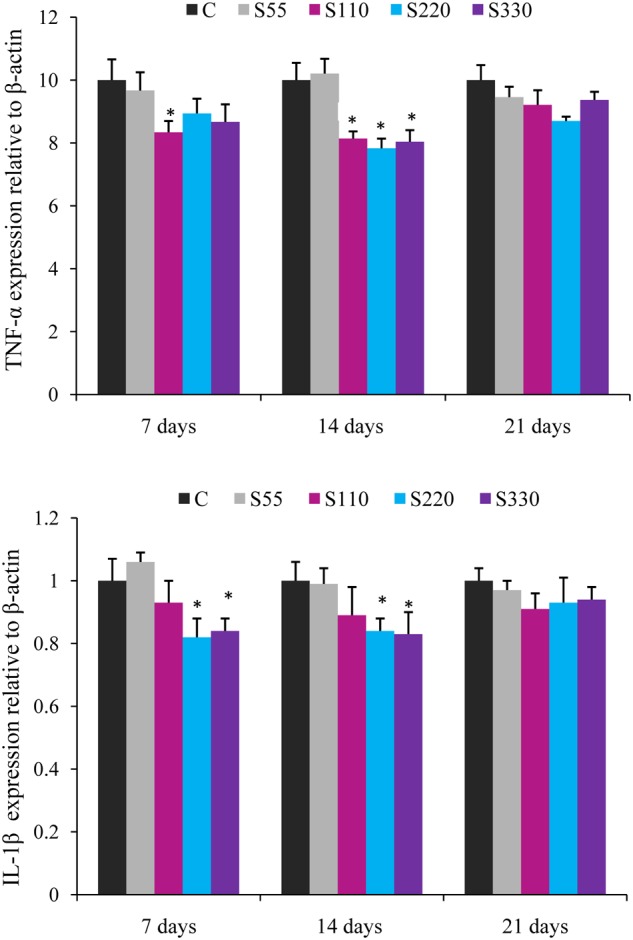
**Relative mRNA expression of pro-inflammatory cytokines (TNF-α, IL-1β) in the head-kidney of *Labeo rohita* administered (i.p.) with biosurfactant.** Bars represent the mean ± SEM (*n* = 9). A significant difference compared to the control is indicated by an asterisk (*P* < 0.05).

**FIGURE 3:**
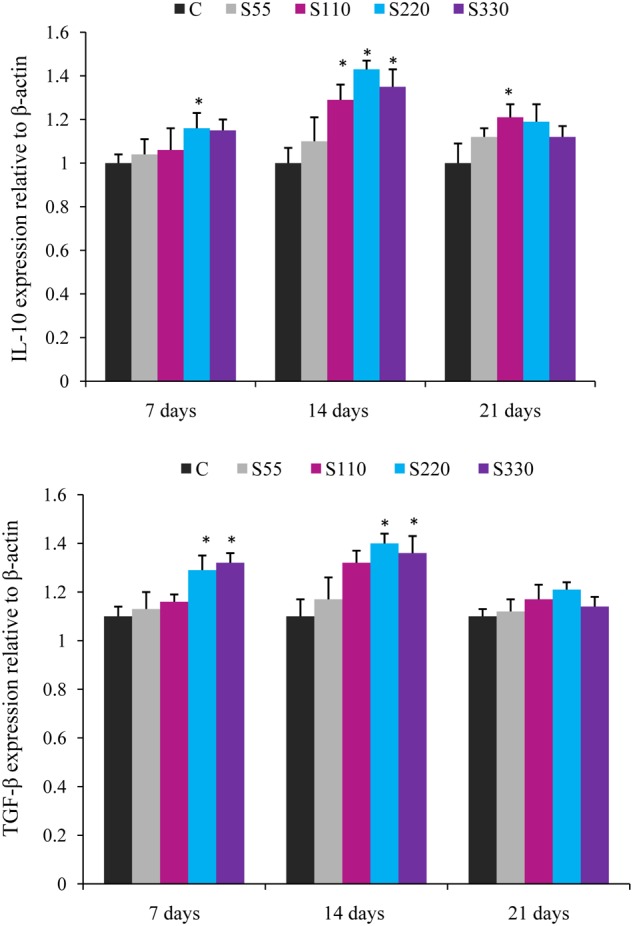
**Relative mRNA expression of anti-inflammatory cytokines (IL-10, TGF-β) in the head-kidney of *L. rohita* administered (i.p.) with biosurfactant.** Bars represent the mean ± SEM (*n* = 9). A significant difference compared to the control is indicated by an asterisk (*P* < 0.05).

**FIGURE 4:**
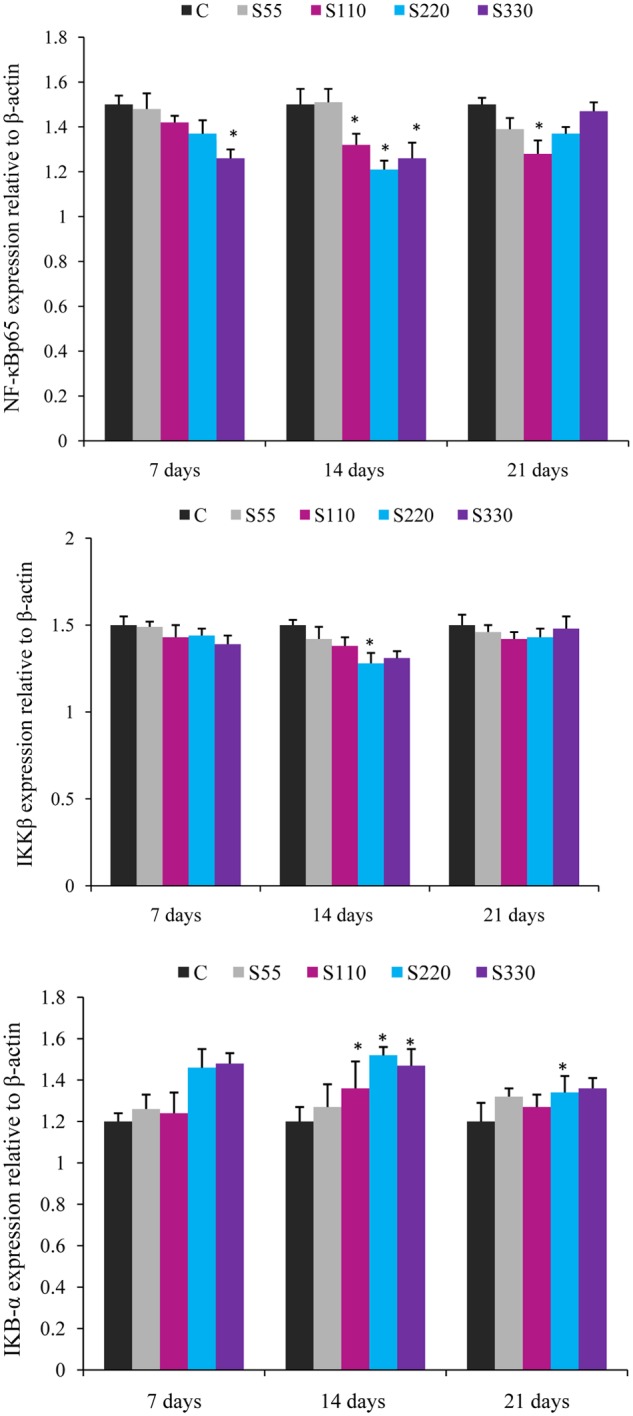
**Relative mRNA expression of cytokines NF-κB p65, IKBα, and IKKβ in the head-kidney of *L. rohita* administered (i.p.) with biosurfactant.** Bars represent the mean ± SEM (*n* = 9). A significant difference compared to the control is indicated by an asterisk (*P* < 0.05).

The expression of cytokine genes in the head kidney of *L. rohita* was altered by biosurfactant administration (i.p.) as shown in **Figures 2**–**4**.

Fish administered biosurfactant (S110–S330) had significantly lower TNF-α levels at 14 dpa (**Figure 2**) when compared to those in the controls, with the lowest levels observed in the S220 group. At 7 dpa, lower (*P* < 0.05) TNF-α expression was recorded in the S330 group. The expression of IL-1β was lower in the treatment groups and differences were significant in the S220 and S330 groups at 7 and 14 dpa (**Figure 2**).

Administration of biosurfactant to fish provoked the expression of anti-inflammatory cytokines IL-10 and TGF-β during the whole experimental period (**Figure 3**). IL-10 expression was higher (*P* < 0.05) in the S110–S330 groups at 14 dpa, with the highest levels in the S220 group. TGF-β mRNA expression was significantly higher in the S220 and S330 groups at 7 and 14 dpa. However, both IL-10 and TGF-β showed a declining tendency at 21 dpa.

The expression of NF-κB p65 showed a tendency to decline in the treated groups at all time points (**Figure 4**). NF-κB p65 expression was significantly lower in the S110–S330 groups at 14 dpa. At 7 and 21 dpa, differences were significant only for the S330 and S110 groups, respectively.

Fish treated with biosurfactant tended to exhibit lower IKKβ expression than those in the control groups at all time points, but the differences were significant only for the S220 group at 14 dpa (**Figure 4**). IκB-α expression was significantly higher in the S110–S330 groups at 14 dpa (**Figure 4**) than in the controls, with the highest expression in the S220 group. At 21 dpa, significantly higher IκB-α expression was recorded in the S220 group.

### Pathogen Challenge Test

The results of the challenge study revealed that fish administered biosurfactant exhibited better protection against *A. hydrophila* infection. Fish administered 220 μg mL^-1^ of biosurfactant (S220) exhibited the highest RPS (72.72%), which was followed by fish in the S330 (57.56%), S110 (39.39%), and S55 (12.11%) groups. No mortality was observed in the negative control group. The lowest survival (8.33%) was recorded in the positive control group.

### Biofilm Inhibition and Heavy Metal Removal Efficiency

*Bacillus licheniformis* VS16-derived biosurfactant was effective in inhibiting biofilm formation, and in removing heavy metals from vegetables (**Table 6**). The biofilm inhibition study showed that the biosurfactant was effective in suppressing biofilm formation by up to 54% (**Table 6A**). Further, the biosurfactant had the potential to remove cadmium from carrots, radishes, ginger, and potatoes (**Table 6B**). Biosurfactant removed 60.98% of Cd from Cd-contaminated ginger.

**Table 6a:** Inhibition of biofilm formation by biosurfactant.

Sample number	Biofilm formation (OD) at 660 nm	Biofilm inhibition by biosurfactant (OD) at 660 nm	% inhibition of biofilm
1	0.316	0.183	41.38 ± 1.09
2	0.239	0.152	36.62 ± 1.13
3	0.388	0.173	54.71 ± 1.27
4	0.281	0.147	47.68 ± 0.54
5	0.226	0.139	38.49 ± 0.68s

**Table 6b:** Initial and final concentrations of cadmium (Cd) in treated vegetables and its removal percent after treatment with biosurfactant.

Sl. No.	Name of the vegetable	Initial concentration of cadmium	Final concentration of cadmium	% Cd removal
1	Carrot	0.5721	0.3427	41.16 ± 1.73
2	Radish	0.5038	0.2396	52.83 ± 1.86
3	Ginger	0.3317	0.1294	60.98 ± 1.29
4	Potato	0.4193	0.2416	42.68 ± 0.74

## Discussion

### Biosurfactant Composition

Biosurfactant production by *B. licheniformis* VS16 was confirmed through several simple yet powerful screening methods such as emulsification activity, drop collapse test, and oil displacement tests. The composition of biosurfactant was characterized through GC–MS analysis (**Figure 1** and **Table 2**). The percentages of peak area were 13.62, 14.3, 18.16, and 21.76% for eicosanoic acid, heptacosane, tert-hexadecanethiol, and hexadecanoic acid, respectively. The highest percentage of peak area was available for hexadecanoic acid and the lowest percentage peak (2.91%) was identified for myristoleic acid at a retention time of 20.93 min. The major abundance of methyl esters of hexadecanoic acid and eicosanoic acid indicated that C_16_ and C_20_ were the main fatty acid components of the purified biosurfactant. The results revealed that the biosurfactant was a mixture of 11 different compounds (C_6_–C_27_) consisting of long chain β-hydroxy fatty acid, hexadecanethiol, and heptacosane. Results obtained in this study were consistent with those of Yakimov et al. (1995), wherein lipopeptide surfactant from *B. licheniformis* BAS50 had a mixture of 14 β-hydroxy fatty acids ranging in size from C_12_ to C_17_. The result was also similar to that of another study by Rajeswari et al. (2016), wherein GC–MS data for a biosurfactant from *S. hominis* showed that it was a mixture of 17 different β-hydroxy fatty acids (C_8_–C_27_).

### Modulation of Immune Responses

The present study demonstrated that *B. licheniformis*-derived biosurfactant was able to modulate immune responses in *L. rohita*. Lysozyme has substantial bactericidal activity against both gram-positive and gram-negative bacteria in the presence of complement (Giri et al., 2015). In fish, complement alarms the host immune system to the existence of potential pathogens and assists in their subsequent clearance (Holland and Lambris, 2002). The highest (*P* < 0.05) LA and ACP activities were recorded at 14 days in the S220 group. However, both activities declined slightly at 21 days, when compared to those obtained at 14 days. Similarly, dietary supplementation with *B. licheniformis* SY-52-derived secondary metabolites was previously shown to enhance LA in *C. carpio* (Chen et al., 2015). Dietary administration of extracellular proteins from *Aeromonas veronii* BA-1 and *Flavobacterium sasangense* BA-3 significantly enhanced lysozyme and complement C3 activities in *C. carpio* (Chi et al., 2014). *L. rohita* administered cellular components of probiotic bacteria had significantly higher LA and ACP activities (Giri et al., 2015). In addition, phospholipid biosurfactants isolated from *S. hominis* increased the LA in *O. mossambicus* (Rajeswari et al., 2016).

‘PA is an important tool and primordial defense mechanism of the non-specific immune system’ (Chen et al., 2015). In the present study, PA was significantly higher in the S220 and S330 groups than in the control group at all time points (**Table 4**). Earlier studies revealed that bacterial metabolites can increase PA in fish (Chen et al., 2015; Giri et al., 2016c). In the present study, IgM levels were higher in the biosurfactant treated groups (**Table 4**). Fish in the S220 group exhibited higher (*P* < 0.05) IgM levels at all time points (**Table 4**). IgM is the predominant antibody type in fish. It is used to identify and neutralize foreign objects such as bacteria and viruses (Giri et al., 2014). Similarly, cellular components of the probiotics *Kocuria* SM1 and *Rhodococcus* SM2 increased IgM levels in *Oncorhynchus mykiss* (Sharifuzzaman et al., 2011). Earlier studies demonstrated that probiotic supplementation increased IgM levels in fish at an initial time point; however, at later time points, these levels declined (Sun et al., 2010; Giri et al., 2013, 2014). In this study, IgM levels increased initially and thereafter they decreased gradually, suggesting that IgM increases is a temporary phenomenon attributable to immunostimulants. Further, LA, PA, ACP, and IgM activities were lower in the S330 group than in the S220 group, suggesting that administration of overdoses of bacterial metabolites could result in the suppression of immune responses in fish (Giri et al., 2016c).

### Digestive Enzyme Activities

Administration (i.p.) of biosurfactant enhanced digestive enzyme activities in fish (**Table 5**). Amylase, protease, and lipase activities were higher in the treated groups. The digestion processes in aquatic animals can be enhanced by the addition of certain microorganisms or their by-products, which can boost the production of extracellular enzymes such as proteases and lipases, and/or can have the intended ability of supplying necessary growth factors as fatty acids and vitamins, among others (Ibrahem, 2015). Earlier studies reported that probiotic bacteria could enhance the secretion of digestive enzymes in fish (Essa et al., 2010; Mohapatra et al., 2012; Sankar et al., 2016). Therefore, increased production of digestive enzymes could improve digestion, which could in turn increase the growth performance of the fish.

### Cytokine Gene Expression

“Cytokines originate from macrophages, lymphocytes, granulocytes, dendritic cells, mast cells, and epithelial cells and include ILs, TNFs, TGFs, IFNs, and chemokines” (Savan and Sakai, 2006). Pro-inflammatory cytokines IL-1β and TNF-α are mainly produced by monocytes and macrophages and regulate numerous aspects of the immune response (Giri et al., 2015). In the present study, administration of biosurfactant (S220 and S330) significantly lowered TNF-α and IL-β expression in the head-kidney of fish at 14 dpa (**Figure 1**). In a similar study, we previously showed that administration of biosurfactant could down-regulate the expression of TNF-α and IL-1β in *L. rohita* (Giri et al., 2016c). However, biosurfactant (S110–S330) significantly upregulated the expression of anti-inflammatory cytokines (IL-10 and TGF-β) at 14 dpa, and maximum expression was recorded in the S220 group (**Figure 2**). This is consistent with increased IL-10 and TGF-β expression observed in *L. rohita* immunized with biosurfactant (Giri et al., 2016c). In addition, IL-10 expression was up-regulated in rainbow trout cell cultures stimulated with lipopolysaccharide (Fierro-Castro et al., 2013) and in *C. carpio* administered bacterial secondary metabolites (Chen et al., 2015). The observed inverse relationships between the expression of pro- and anti-inflammatory cytokines in the present study is consistent with earlier studies wherein fish were administrated immunostimulants (Feng et al., 2016; Giri et al., 2016c). IL-10 is produced mainly by monocytes, macrophages, B cells, T cells, and dendritic cells. It inhibits the expression of many pro-inflammatory cytokines (De Waal Malefyt et al., 1991). “TGF-β inhibits B and T cell proliferation and differentiation, antagonizes pro-inflammatory cytokines such as IL-1β, TNF-α, and IFN-γ, and blocks the expression of IL-1β and IL-2 receptors” (Li and Flavell, 2008). These results suggest that the administration of 220 μg mL^-1^ of biosurfactant attenuates pro-inflammatory responses in fish.

NF-κB, a critical transcription factor that is involved in the inflammatory response, controls the expression of various compounds such as IL-6, IL-1β, TNF-α, iNOS, and COX-2 (Cho et al., 2013). In this study, mRNA expression of NF-κB p65 significantly declined during the entire duration of the trial (**Figure 3**) and the lowest expression was recorded in the S220 group at 14 dpa. Inhibition of NF-κB was previously shown to down-regulate the expression of IL-1β and IL-8 in mouse monocytes (Li et al., 2008). Further, IκB-α expression was higher in the biosurfactant-treated groups (**Figure 3**). However, IKK-β mRNA was lower in the treated groups and this difference was significantly lower only in the S220 group at 14 dpa (**Figure 3**). Up-regulation of IκB-α, an NF-κB binding protein, was shown to lead to down-regulation of pro-inflammatory cytokines in mouse muscle fibers (Hnia et al., 2008). Further, IL-10 can attenuate pro-inflammatory cytokine production through the suppression of NF-κB activation by sustaining IκB protein expression (Chon et al., 2010). Therefore, our results indicated that biosurfactant might act by down-regulating the expression of IKK-β to attenuate the degradation of IκB-α, thereby inhibiting NF-κB p65 nuclear translocation, to suppress the expression of pro-inflammatory cytokines (Giri et al., 2016c). However, the underlying mechanism requires further investigation.

### Challenge Study

In the present study, carp treated with (i.p.) biosurfactant showed increased protection against *A. hydrophila*. Fish administered 220 μg mL^-1^ of biosurfactant (S220) exhibited the highest RPS (72.72%). In a recent study, we demonstrated that *L. rohita* treated with (i.p.) with 200 μg mL^-1^ of *B. subtilis*-derived biosurfactant were most protected against *A. hydrophila* infection (Giri et al., 2016c). Dietary administration of secondary metabolites isolated from *B. licheniformis* XY-52 enhanced the survival of *C. carpio* against *A. hydrophila* challenge (Chen et al., 2015). *O. mossambicus* administered *S. hominis*-derived phospholipid biosurfactant (200 mg kg^-1^) were most protected against *A. hydrophila* challenge (Rajeswari et al., 2016). The improved immune parameters (LA, ACP, PA, and IgM) and digestive enzyme activities, and higher expression of anti-inflammatory cytokines in fish treated with 220 μg mL^-1^ of biosurfactant might be linked with the enhanced resistance to *A. hydrophila*, which could result in higher post-challenge survival. The groups injected with 55, 110, or 330 μg mL^-1^ of biosurfactant exhibited lower post-challenge survival, which might be associated with the obtained results regarding immune responses, digestive enzyme activities, and immune gene expression. These results indicate that *B. licheniformis* VS16-derived biosurfactant has immunomodulatory effects on carp.

### Biofilm Inhibition and Heavy Metal Removal by Biosurfactant

The biosurfactant was effective in suppressing biofilm formation (**Table 6A**) by up to 54%. Controlling the adherence of microorganisms to the food contact surface is critical for providing safe and healthy food products to consumers (Anjum et al., 2016). Therefore, the use of biosurfactants in inhibiting biofilm formation could be an important tool for the food industry. Glycolipid biosurfactant (30 μg glycolipid mL^-1^) derived from *Brevibacterium casei* was shown to significantly inhibit biofilm production by *Vibrio* spp., *E. coli*, and *Pseudomonas* spp. of both mixed culture and individual strains (Kiran et al., 2010a). *Nocardiopsis* sp. MSA13A-derived biosurfactant (300 μg mL^-1^) significantly disrupted biofilm formation in *Vibrio alginolyticus* (Kiran et al., 2014). Biosurfactant isolated from *Lysinibacillus fusiformis* S9 exhibited notable inhibition of biofilm formation by pathogenic *E. coli* or *Streptococcus mutans* (Pradhan et al., 2014). They reported that at a concentration of 40 μg mL^-1^ the biosurfactant efficiently inhibited biofilm formation *by E. coli* and *S. mutans.* Further, Falagas and Makris (2009) hypothesized that ‘probiotics or their products (biosurfactants) could be applied to patient care equipment such as tubes or catheters with the aim of decreasing colonization by nosocomial pathogens.’

The intake of heavy metals though food or any other means is highly detrimental to human health. The uptake of heavy metals by crops is often associated with the plant species, growth phase, soil type, metal variety, soil conditions, environment, and weather. We found that biosurfactant has the potential to remove cadmium from carrots, radishes, ginger, and potatoes (**Table 6B**). Recently, Anjum et al. (2016) demonstrated that *Bacillus* sp. METCC 5877-derived biosurfactant removes considerable amounts of heavy metals from vegetables such as potatoes, garlic, radishes, and onions. In addition, rhamnolipids (glycolipids) removed nickel and cadmium from soils with efficiencies of 80–100%, when tested in a controlled environment, and this removal efficiency was 20–80%, when using field samples (Wang and Mullingan, 2004). Therefore, the controlled and cautious use of these exciting surface active molecules will definitely assist in the removal of the toxic environmental pollutants and could help provide a clean environment.

## Conclusion

Injection (i.p.) of *L. rohita* with 220 μg mL^-1^ biosurfactant derived from *B. licheniformis* VS16 boosted immune responses, leading to improved functional immunity in terms of disease resistance against live virulent *A. hydrophila*. Furthermore, biosurfactant administration modulated cytokine-related gene expression in the head kidney of fish. Biosurfactants are biocompatible and biodegradable, and were shown to effectively inhibit biofilm formation and efficiently remove heavy metals from tested vegetables. Therefore, purified biosurfactant from *B. licheniformis* VS16 could have applications in aquaculture as an immunostimulant to prevent diseases, as well as in food processing for the removal of heavy metals from vegetables and to control biofilm formation.

## Author Contributions

Designed the experiments: SG, VS. Conceived and supervised the study: SS, VS, SP. Performed the experiments: SG, SS, VS. Data analyzed by: SG, JJ. Wrote the manuscript: SG, SS. Proofread the manuscript: SP.

## Conflict of Interest Statement

The authors declare that the research was conducted in the absence of any commercial or financial relationships that could be construed as a potential conflict of interest.

## Footnotes

1 https://www.ncbi.nlm.nih.gov/blast/
